# Hematochezia: An Uncommon Presentation of Colonic Tuberculosis

**DOI:** 10.1155/2017/7831907

**Published:** 2017-04-03

**Authors:** Fares Ayoub, Vikas Khullar, Harry Powers, Angela Pham, Shehla Islam, Amitabh Suman

**Affiliations:** ^1^Department of Medicine, University of Florida, Gainesville, FL 32608, USA; ^2^Department of Medicine, Division of Gastroenterology, University of Florida, Gainesville, FL 32608, USA; ^3^Department of Medicine, Division of Infectious Disease, University of Florida, Gainesville, FL 32608, USA

## Abstract

Abdominal tuberculosis (TB) is an uncommon entity in the United States. Colonic TB is reported in 2-3% of patients with abdominal TB. It is frequently misdiagnosed as Crohn's disease or carcinoma of the colon due to their shared clinical, radiographic, and endoscopic presentations. We present a case of a 72-year-old male with colonic tuberculosis presenting as hematochezia. Our patient presented with shortness of breath and weight loss. Chest X-ray demonstrated ill-defined bilateral parenchymal opacities in the perihilar, mid, and lower lung zones. The patient was diagnosed and treated for community acquired pneumonia, with no improvement. Hematochezia complicated by symptomatic hypotension developed later in the course of admission. Colonoscopy revealed multiple ulcers at the anus and transverse and ascending colon as well as the cecum with stigmata of bleeding. Biopsy of a sigmoid ulcer was consistent with colonic tuberculosis. Antitubercular therapy was initiated, but the patient passed away secondary to multiorgan failure 29 days into admission.

## 1. Introduction

After a resurgence in the incidence of tuberculosis (TB) infections in the United States between 1985 and 1992 due to the human immunodeficiency virus (HIV) epidemic, the incidence of TB had annually declined. However, as of 2015, TB incidence has leveled in the US and TB elimination (defined as <1 TB case per 1 million persons annually) remains elusive [1]. Gastrointestinal tuberculosis is a manifestation of extrapulmonary tuberculosis. In 2014, extrapulmonary TB constituted 20.57% of TB cases reported to the CDC and continues to be a missed diagnosis for providers not considering TB in their differential diagnoses [2].

Tuberculosis affecting the gastrointestinal tract was recognized as early as the fourth century BC in texts by Hippocrates [3]. While TB of the gastrointestinal tract is not as common as pulmonary TB, it is an important cause for TB related morbidity and mortality. The pathophysiology of this form of tuberculosis involves spread of mycobacteria to the gastrointestinal tract by number of means: hematogenous spread, swallowing of sputum contaminated with live* M. tuberculosis* bacilli, ingestion of contaminated food, or direct spread from adjacent organs [4]. The terminal ileum and the ileocecal valve are the most commonly affected parts, followed by the ascending colon which is usually affected through continuous involvement extending from the cecum. This predilection for the terminal ileum has been attributed to the presence of large amounts of lymphoid tissue and the longer contact duration of gastrointestinal contents with the lumen [5].

Classically, gastrointestinal TB may present with fever, weight loss, anorexia, abdominal pain, nausea, vomiting, or diarrhea. Hematochezia is a less common presentation [5]. Physical exam findings are nonspecific but may include abdominal tenderness, ascites, and hepatomegaly [4, 5]. Diagnosis is often delayed, as this form of tuberculosis is commonly misdiagnosed as Crohn's disease or carcinoma of the colon due to their similar clinical, radiographic, and endoscopic presentations [5, 6]. Laboratory testing is also nonspecific but may reveal anemia, leukocytosis, increased alkaline phosphatase, and hypoalbuminemia. A chest X-ray may demonstrate evidence of pulmonary TB; however, a normal reading does not exclude disease, as only 15–20% of intestinal TB is associated with active pulmonary TB [7]. CT scanning of the abdomen can exhibit mural thickening, extramural inflammation, and strictures [8]. Colonoscopy can demonstrate ulceration, nodularity, polyps, and luminal narrowing. Mukewar et al. found ulceration to be the most common lesion, found in 88% of patients. Ulcers were largely linear, transverse, or circumferential, covered with yellowish or whitish exudates and surrounding mucosa was inflamed with edema and nodularity. Other lesions found on colonoscopy include polyps which mimicked carcinoma of the colon, as well as unnegotiable luminal narrowing in a smaller subset of patients [5]. Biopsy of colonic ulcers typically demonstrates either caseating or noncaseating granulomas with predominantly lymphocytic chronic inflammation. Acid-fast bacilli (AFB) staining can demonstrate the presence of mycobacteria; however, in one series, this was reported to be positive in only 36% of cases. Other diagnostic tests include tissue culture, PCR, and immunostaining [9].

Treatment of gastrointestinal tuberculosis is analogous to the treatment of pulmonary TB, with 2 months of conventional antituberculous therapy (rifampicin, isoniazid, pyrazinamide, and ethambutol), followed by rifampicin and isoniazid for an additional 4 months [10]. Surgical resection may be required in cases of severe stricture causing high-grade intestinal obstruction.

## 2. Case

A 72-year-old African American male with no past medical history was hospitalized for shortness of breath and unintentional weight loss for a month prior to presentation. On further questioning, he reported that his shortness of breath had developed over the past year and was associated with an intermittent dry cough. A few days prior to presentation, he had developed fatigue, subjective fever, and chills. He was otherwise asymptomatic. At the time of presentation, he had a blood pressure of 134/85 mmHg, a heart rate of 128 bpm, a respiratory rate of 18 bpm, and an oral temperature of 37.1°C. On physical examination, he appeared to be cachectic. His chest and abdominal exams were within normal limits. The remainder of the examination was unremarkable.

**Figure 1:**
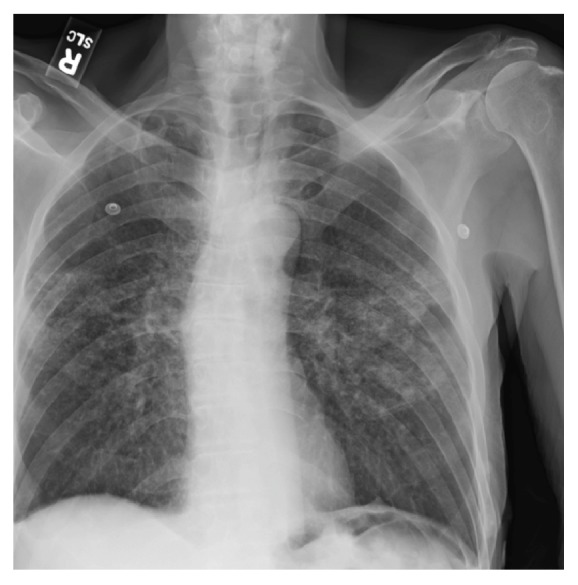
Posteroanterior chest X-ray demonstrating extensive ill-defined bilateral parenchymal opacities in the perihilar, mid, and lower lung zones.

Laboratory testing revealed a myriad of abnormalities. A complete blood count was significant for normocytic anemia with a hemoglobin of 12 g/dL and an MCV of 81.0 fL as well as thrombocytosis with a platelet count of 552 × 10^3^/microliter. A metabolic panel revealed hyponatremia with a sodium level of 123 mmol/L, hyperkalemia with a potassium of 6.2 mmol/L, acute renal failure with a creatinine of 8.0 mg/dL and a BUN of 98 mg/dL, and an elevated anion gap metabolic acidosis with a pH of 7.3, a serum CO2 of 11 mmol/L, and an anion gap of 28 mmol/L. Testing for human immunodeficiency virus (HIV) was negative. Urinalysis was remarkable for pyuria with >180 WBC/high powered field and a negative urine nitrite. This was thought to indicate sterile pyuria, as conventional urine culture techniques were initially negative. A chest X-ray revealed extensive ill-defined bilateral parenchymal opacities in the perihilar, mid, and lower lung zones (Figure 1). The patient was admitted to the hospital for further management of acute renal failure and respiratory abnormalities. Blood and urine cultures were obtained. The patient was hydrated with intravenous normal saline and intravenous ceftriaxone therapy was initiated for a tentative diagnosis of community acquired pneumonia. Over the course of the following three days, the patient's hemoglobin decreased from 12 g/dL to 8 g/dL. A fecal immunohistochemical test for blood in stool was positive and the gastroenterology consult service recommended both an upper endoscopy and lower endoscopy.

**Figure 2:**
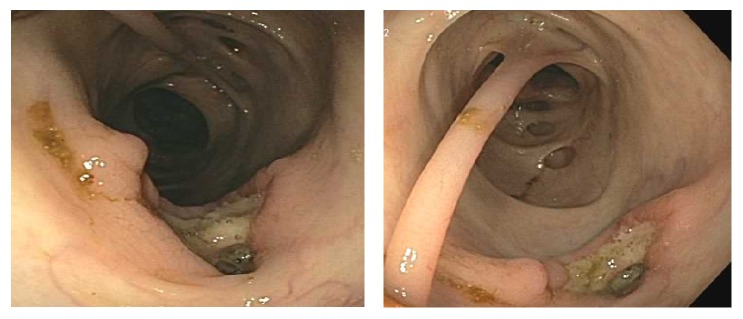
First colonoscopy: extensive diverticulosis and solitary ulcer in the sigmoid colon.

**Figure 3:**
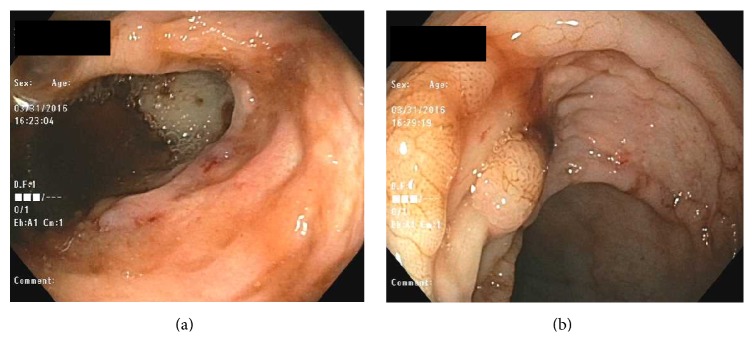
Second colonoscopy: (a) cecal ulcer and (b) transverse colon ulcer.

Esophagogastroduodenoscopy was unremarkable. Colonoscopy was marred by inadequate bowel preparation but was significant for diverticulosis in the sigmoid and descending and transverse colon with purulent discharge associated with one diverticular opening. It also demonstrated an ulcer in the sigmoid colon which was biopsied (Figure 2). The presence of other ulcers was unclear due to the poor bowel preparation. These findings raised suspicion for acute diverticulitis and the patient's antimicrobial coverage was broadened to include ciprofloxacin and metronidazole. The following day the patient passed a large amount of blood per rectum, developed hypotension with a blood pressure of 79/49 mmHg, and was transferred to the medical intensive care unit. A repeat urgent colonoscopy revealed multiple ulcers at the anus and transverse and ascending colon as well as the cecum; the terminal ileum appeared normal (Figure 3).

**Figure 4:**
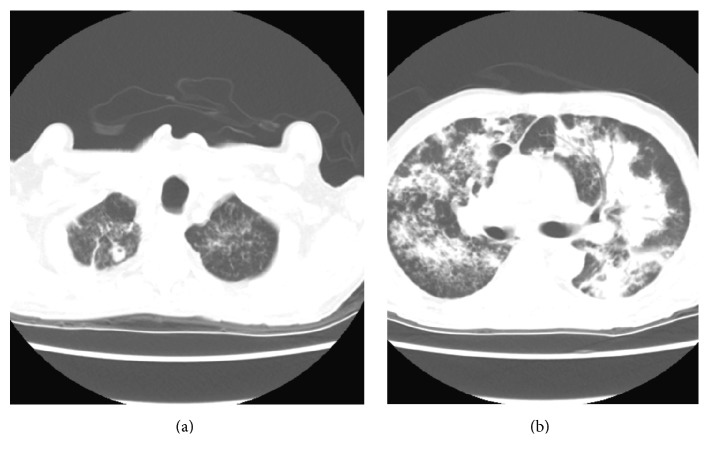
CT chest with IV contrast taken on day 13. (a) shows right upper lobe cavitary lesion. (b) shows multilobar consolidation.

**Figure 5:**
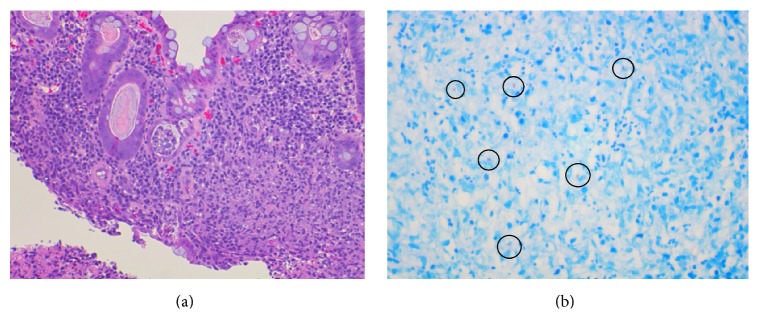
(a) 20x hematoxylin and eosin (H&E) stain demonstrating active colitis with crypt abscess and an ill-defined defined granulomatous area. (b) 40x AFB stain of the splenic flexure with necrotizing granuloma and acid-fast bacilli (circled).

A CT scan of the chest obtained for acute hypoxic respiratory failure showed extensive patchy consolidation and reticulonodular opacities throughout the lungs with a 1.3 cm cavitary lesion in the right apex as well as mediastinal and hilar lymphadenopathy. These findings were suspicious for atypical infection (Figure 4). The patient underwent bronchoscopy with bronchoalveolar lavage and a lavage sample was AFB stain positive. Pathology of the sigmoid ulcer biopsy revealed focal active colitis with cryptitis, crypt abscesses, and mild stromal lymphoplasmacytic inflammation. AFB stain of the sigmoid ulcer biopsy was positive with findings consistent with mycobacterium (Figure 5). AFB stain of a urine sample obtained to explain sterile pyuria was also positive. The patient was placed under respiratory isolation and was started on appropriate antitubercular therapy.

Over the course of our patient's hospitalization, his clinical condition continued to decline. Multiorgan failure requiring vasopressor support later ensued and the patient ultimately passed away 29 days into admission.

## 3. Discussion

We present a case of diffuse systemic tuberculosis (TB) infection involving the colon, urinary tract, and lungs complicated by multiorgan failure and death. Unique to our patient was the lack of typical risk factors for TB infection. The patient had no history of travel to TB endemic areas and denied incarceration, homelessness, or exposure to infected individuals. It remains unclear where the patient may have contracted the infection; however, earlier exposure to infected individuals or family members that the patient does not recall is possible. Also unique in our patient's case is the presentation of colonic tuberculosis with hematochezia causing hemodynamic instability. Rectal bleeding is a rare presentation of colonic TB; only a handful of case reports have documented this manifestation [11].

Our patient's initial colonoscopy findings of a rectal ulcer, as well as diverticulosis showing signs of active infection, were highly suspicious for acute diverticulitis (Figure 2). However, initial colonoscopy was marred by poor bowel preparation and did not demonstrate the extent of colonic involvement with ulceration and there was low suspicion of active tuberculosis as a potential cause for the patient's drop in hemoglobin. Given our patient's advanced age as well as the presence of multiple diverticulae, angiodysplastic changes in colonic vessels were also part of our differential for lower gastrointestinal bleeding. However, massive hematochezia the following day prompted repeat colonoscopy which showed multiple new ulcers involving all segments of the colon, raising concern for an infectious process (Figure 3). No biopsies were obtained during the second colonoscopy due to active bleeding. Acid-fast stain of the sigmoid ulcer biopsy obtained during the first colonoscopy showed acid-fast organisms consistent with mycobacteria (Figure 5).

Tuberculosis remains a “great mimicker” and must continue to be part of the differential diagnosis in cases of colitis especially in individuals who have additional findings suspicious for infection on chest imaging. While staining for acid-fast bacilli is not routine practice in pathology laboratories, this should be considered in select patients who present with concerns for infection, especially both in the lungs and in the gastrointestinal tract. As described above however, only 15–20% of intestinal TB is associated with active pulmonary TB, thus providers must maintain a high index of suspicion to allow early diagnosis and management.

Our case also serves as a reminder for the importance of postexposure testing and prophylaxis in healthcare workers. Nosocomial transmission of TB in the healthcare setting is well described; cases of multidrug resistant TB transmitted to providers and patients in hospitals have also been reported [12, 13]. In hospitals that receive more than 200 TB admissions a year, incidence of hospital worker infection has been reported to be as high as 10% a year [14].

Hospital-based infection control programs are essential in the control of such transmission. Airborne infection isolation rooms (previously termed negative pressure isolation rooms) should be utilized for patients with suspected or confirmed tuberculosis. N95 masks that filter particles ≥1 micrometer in diameter with at least 95 percent efficiency should be utilized by healthcare staff and offered to visitors of patients with suspected or confirmed tuberculosis. Nonessential invasive diagnostic procedures ought to be postponed unless absolutely necessary [15].

Providers exposed to patients with active pulmonary tuberculosis should be assessed for symptoms suggestive of active TB infection (hemoptysis, weight loss, and fever) and, if present, should undergo chest X-ray with sputum acid-fast stain and culture. Providers without symptoms of active infection are to undergo tuberculin skin testing or an interferon-gamma release assay at baseline and again 8 to 12 weeks postexposure [12].

In our case, all providers who may have been exposed to the patient underwent interferon-*γ* release assay testing (QuantiFERON, Cellestis Ltd., Australia) at baseline and 8 weeks postexposure. The patient's family members were also contacted and offered screening and appropriate treatment for TB.
